# From Bytes to Bedside: Data Integration and Computational Biology for Translational Cancer Research

**DOI:** 10.1371/journal.pcbi.0030012

**Published:** 2007-02-23

**Authors:** Jomol P Mathew, Barry S Taylor, Gary D Bader, Saiju Pyarajan, Marco Antoniotti, Arul M Chinnaiyan, Chris Sander, Steven J Burakoff, Bud Mishra

**Affiliations:** National Center for Biotechnology Information, United States of America

Major advances in genome science and molecular technologies provide new opportunities at the interface between basic biological research and medical practice. The unprecedented completeness, accuracy, and volume of genomic and molecular data necessitate a new kind of computational biology for translational research. Key challenges are standardization of data capture and communication, organization of easily accessible repositories, and algorithms for integrated analysis based on heterogeneous sources of information. Also required are new ways of using complementary clinical and biological data, such as computational methods for predicting disease phenotype from molecular and genetic profiling. New combined experimental and computational methods hold the promise of more accurate diagnosis and prognosis as well as more effective prevention and therapy.

## Introduction

Over the last two decades, our knowledge of cancer and its causes has increased greatly. However, we still have few examples of cures. This underscores the need for a clearer understanding of the alterations in the biological circuitry that lead to tumor development and growth. Sequencing of the human genome and biotechnological advances have led to the generation of large volumes of genome-scale data. Combining this genome-scale molecular data with clinical information provides new opportunities to discover how perturbations in biological processes lead to disease. This knowledge can be used to improve disease diagnosis, prognosis, prevention, and therapy. However, the large scale and diversity of both experimental and clinical data necessitate that they be well-organized and computationally accessible to research scientists for analysis and interpretation. This review focuses on the challenges and opportunities to combine clinical and genome-scale molecular data, using computational approaches, to better understand cancer biology and to translate this knowledge into improved disease prevention and therapy.

### 

#### Translational cancer research to improve disease prevention and therapy.

Cancer is a complex disease, involving multiple and specific changes at the DNA level that can be inherited or induced by environmental factors. There are many different types and subtypes of cancer marked by specific sets of molecular changes. Most of our current cancer treatment efforts are focused on surgery for a curative treatment and radiation and/or toxic drugs (chemotherapy) to induce remissions. Candidates for successful cancer therapy with surgery are few, and radiation and chemotherapy suffer from lack of target specificity, leading to serious side effects. Identifying cancer-specific molecular changes and discovering how they can be used to increase therapeutic specificity will lead to higher success rates and fewer side effects.

Translational cancer research seeks to identify and understand the cause and effect of cancer-specific molecular defects and to translate this “bench” knowledge to the clinic to improve disease prevention and therapy. Examples of research questions include, from a clinical perspective: what are the molecular subtypes of cancer? What reliable molecular markers are available for early cancer detection (diagnostic) and for predicting the course of disease (prognostic)? How do we find better drugs and optimize therapy (development of more specific drugs with lower toxicity) to suit an individual patient's molecular profile? From a molecular biology perspective: can we accurately predict vulnerable point(s) in molecular pathways that are potential therapeutic targets? What specific drug or drug combination can target these vulnerable points in the pathway? Can genotype and pathway information be combined to predict the effect of a mutation on disease or therapy?

Advances in our understanding of cancer-specific molecular defects have led to improved cancer treatments. For example, the protein kinase inhibitor imatinib (Gleevec) was designed to treat chronic myelogenous leukemia (CML) based on knowledge of the causative molecular defect—translocation and dys-regulated BCR–ABL kinase. Protein kinase inhibitors such as gefitinib (Iressa) and erlotinib (Tarceva) are showing therapeutic promise by targeting known molecular abnormalities of non-small cell lung cancer (NSCLC). Similarly, antibody therapies such as Rituximab (Rituxan), an anti-CD20 monoclonal antibody for non-Hodgkin lymphoma; Cetuximab (Erbitux), an epidermal growth factor receptor (EGFR)-binding antibody for colorectal and head and neck cancer; Trastuzumab (Herceptin), a monoclonal antibody that allows targeted therapy in HER2 positive breast cancer; and Bevacizumab (Avastin), a recombinant humanized antibody against vascular endothelial growth factor (VEGF) for metastatic colorectal cancer are promising.

While these treatments based on molecular knowledge of the cancer show promise, major challenges remain. For instance, development of compensatory mutations induces resistance to Gleevec and limits its use, while humanization and effective delivery of antibodies is difficult [1]. Furthermore, the discovery and development of a new and effective drug can cost US$0.8–US$1.7 billion [2]. A new drug entering Phase I testing, where the drug is initially introduced to human subjects, is estimated to have only an 8% chance of reaching the market [2]. Failures can largely be attributed to poor target selection or poor candidate drug selection, leading to low drug effectiveness or toxicity. Development of safe and effective therapies, such as small molecule protein kinase inhibitors, at a reduced cost, requires better understanding of therapeutic interaction of the inhibitor with a range of targets and their effect on diverse cellular processes [3].

**Figure 1:**
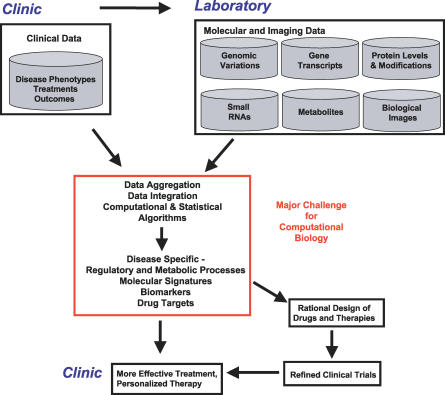
Data Integration for Translational Cancer Research Archival of clinical and molecular data in easily retrievable standardized formats, aggregation, integration, and data analysis will provide opportunities for the next-generation biomedical discoveries that can impact cancer research and treatment.

Cancer cells can now be profiled on a genome scale using new experimental techniques. We thus have an unprecedented opportunity to comprehensively study cancer-specific molecular processes. This study requires computational tools to handle the large volume and diversity of available information. Collection, standard organization, aggregation, storage, integration, and analysis of diverse genome-scale molecular data along with patient data collected in the clinic will broaden our understanding of how cancer-specific molecular defects affect clinical outcome and will lead to improved disease prevention and therapy (Figure 1).

### Genome-Scale Molecular Data for Cancer Research

#### Genomic variation.

Cancer is a genetic disease involving point mutations, translocations, segmental amplifications, and deletions in the genome that alter specific vulnerable molecular points in cellular regulatory pathways. Analysis of chromosomal changes by fluorescent in situ hybridization (FISH)-based cytogenetic approaches including comparative genomic hybridization (CGH), spectral karyotyping (SKY), and multiplex-FISH (M-FISH) [4] have led to the characterization of many cancer-associated chromosomal abnormalities. Microarray techniques, e.g., array-CGH or matrix-CGH, have become available to map regions of DNA sequence from the cancer tissue that are amplified or reduced compared to normal tissue [5]. Array-based technologies also allow genome-wide measurement of single nucleotide polymorphisms (SNPs) [6]. The international HapMap project has identified millions of SNPs in different populations. The data has been processed into haplotypes, sets of co-occurring SNPs, and tag SNPs (SNPs that distinguish a set of common haplotypes) that can be used to reduce the complexity of gene association studies (http://www.hapmap.org) [7].

Epigenetic changes such as DNA methylation, histone modification, and RNA silencing are involved in regulating many cellular processes, including development, via gene silencing (chromatin structure and transcription regulation) and genetic imprinting. Specific DNA methylation alterations have been identified in various neoplasms. For example, aberrant promoter methylation associated with transcriptional downregulation of tumor suppressor genes has been found in basal cell carcinoma (BCC), cutaneous squamous cell carcinoma (SCC), melanoma, and cutaneous lymphoma [8]. Though not exhaustive, Table 1 gives a list of publicly available cancer-relevant large genomic variation repositories.

**Table 1:**
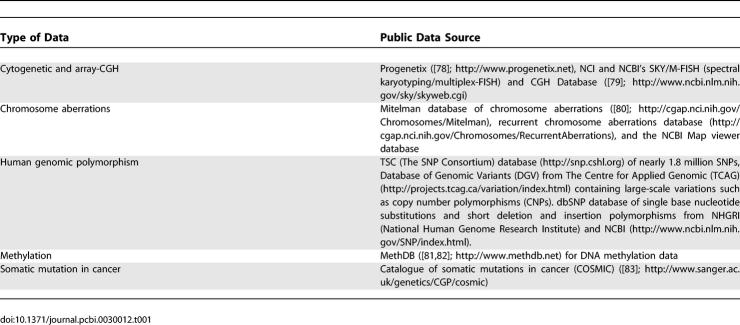
Genomic Variation Repositories

Several projects attempt to comprehensively study genomic variation in cancer. The Cancer Genome Atlas (http://cancergenome.nih.gov/index.asp) and the Sanger Institute's Cancer Genome Project (http://www.sanger.ac.uk/genetics/CGP) aim to identify mostly somatic mutations in common tumor types using next generation DNA sequencing technology.

#### Gene transcript profiles.

Global gene expression profiling with DNA microarrays [9–11] has furthered our understanding of the regulation of biological processes and has become an indispensable tool in the study and classification of human tumors. Semiquantitative profiles of gene expression have been measured for many cancer types and subtypes [12,13]. Through unbiased comparative analysis of these profiles, a subset of genes can be found that correlate with tumor phenotype and can serve as diagnostic and prognostic markers of disease. Disease-specific regulatory programs can be studied using techniques such as chromatin immunoprecipitation (ChIP) of tumor biopsies [14]. Several large public compendiums of gene expression data generated from diverse experimental methods also exist, some examples of which are presented in Table 2.

**Table 2:**
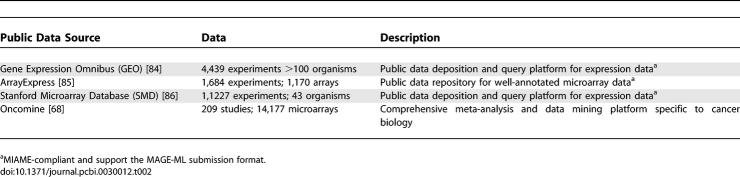
Gene Expression Repositories

Not all available cancer microarray data are generated from gold-standard tissues or primary cell culture. Often, immortalized cell line models of neoplastic disease are studied because they are easier to access than histopathologically characterized human tumors [15,16]. However, in vitro extended passaging of cell lines could lead to accumulation of alterations and yield less representative expression profiles and less stable disease phenotypes [17]. Additionally, the in vivo microenvironment affects tumor–host interactions and causes variations in gene expression and pathways. These changes make disease-specific expression patterns difficult to infer from cell line data alone. Nevertheless, mining and analysis of the large amount of available cell line gene expression data promises new insights into disease conditions.

#### Protein levels and modifications.

**Figure 2:**
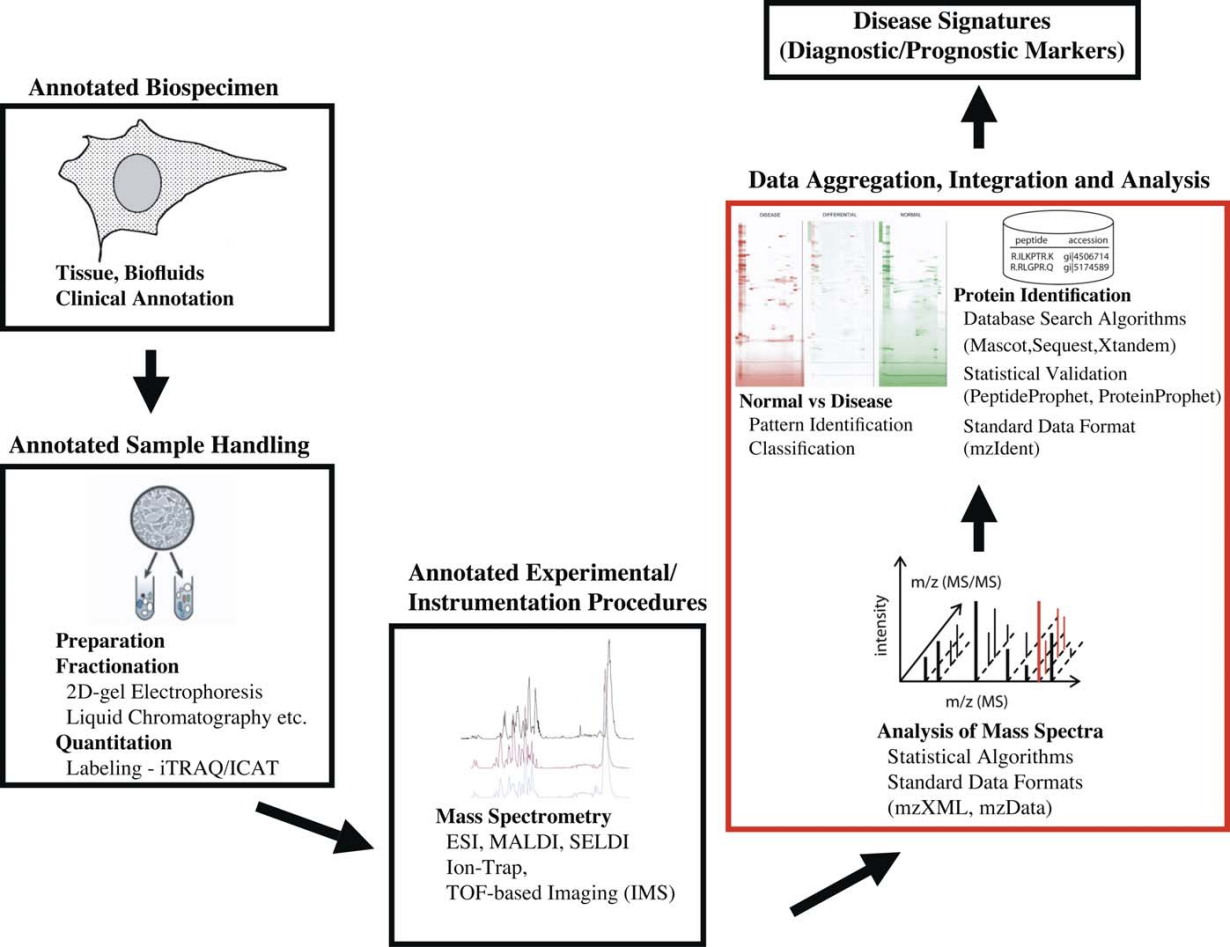
Protein Profiling for Cancer Diagnosis and Prognosis Generation of protein profiles using mass spectrometry is an example of an experimental technique that produces massive amounts of data that is difficult to interpret without computational and statistical algorithms. For instance, comparison of disease versus control sample profiles can lead to identification of disease-specific protein expression signatures, which could be used as diagnostic or prognostic markers. Aggregation of such data from multiple sources and pooled analysis requires proper annotation of sample source, sample handling, and experiment information.

Mass spectrometric instruments and protein chip technology allow large-scale analysis of proteins, their quantitative expression, interactions, post-translational modifications, and localization [18–25]. Proteomic profiling of clinical samples ranging from tissues to biofluids (e.g., urine, sera, plasma, whole blood, cerebrospinal fluid, and saliva) will help assess disease development and progression, generate diagnostic and prognostic disease markers, and predict patient response to intervention. This information can be used to identify regulatory networks and activated signaling events in biological pathways, and can help characterize pathologically benign and tumor tissue samples (Figure 2). Public proteomics repositories that collect this data are now available, and relevant examples are listed in Table 3. Accurate protein identification involving processing and identification of mass spectral peaks, peptide sequencing, search algorithms, and statistical validation of correct assignment of peptides and proteins is challenging, often confounded by splice variants or other protein isoforms [26–30].

**Table 3:**
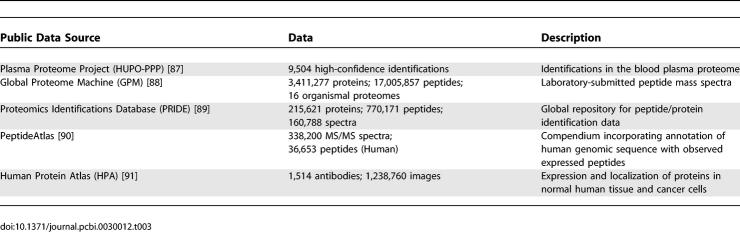
Proteomics Repositories

#### Small RNAs.

Small RNAs add an additional layer of complexity to gene regulation [31]. Initially discovered in plants and *C. elegans,* at least four subfamilies exist: microRNAs (miRNAs), short interfering RNAs (siRNAs), tiny noncoding RNAs (tncRNAs), and small modulatory RNAs (smRNAs). miRNAs are small, ∼21–24, nucleotide noncoding endogenous RNAs involved in translational repression and messenger RNA (mRNA) cleavage, which can potently downregulate translation of specific mRNAs by targeted 3′-UTR binding. An increasing number of miRNAs have been implicated in disease. For instance, a small cluster of miRNA genes on Chromosome 13, c13orf25, appears to be involved in B cell lymphoma [32]. Cell cycle regulation has been shown to involve a miRNA regulatory circuit where *c-Myc,* a known proto-oncogene upregulating E2F1 (a cell cycle regulator), also regulates the expression of six miRNAs on Chromosome 13, two of which have been shown to downregulate E2F1 [33]. In the case of acute lymphoblastic leukemia (ALL), in studies by Lu et al. (2005), miRNA transcriptional profiles have been shown to better classify tumors by type and developmental stage than mRNA profiles [34].

mRNA targets for several hundred miRNAs that are expressed in human have been computationally predicted, though few targets have been experimentally confirmed ([35,36], http://www.microrna.org). The lack of perfect base-pair complementarity between the miRNA sequence and its target and the short length of the miRNAs make accurate prediction of miRNA genes and targets difficult.

#### Pathways.

Pathway information is vital for understanding biological processes and how they are disrupted or reprogrammed in disease. However, collecting complex pathway information in a usable form from diverse and heterogeneous sources, including more than 220 pathway databases (http://pathguide.org), is a major challenge [37]. A number of pathway database efforts seek to ameliorate the situation by making pathway data more accessible for computational analysis (Table 4). For instance, Memorial Sloan-Kettering Cancer Center and the Institute of Bioinformatics have collaboratively developed ten large cancer-focused signaling pathways (http://cancer.cellmap.org).

**Table 4:**
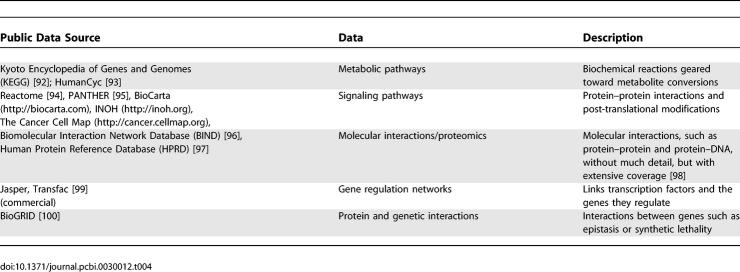
Human-Focused Pathway Repositories

#### Metabolite profiles.

Metabolomics involves measurement of metabolite concentrations and fluxes in cells and tissues [38]. Such measurements provide insight into the response of biological systems to genetic and environmental influences. Metabolites provide important markers for disease state and the pathways underlying drug metabolism. Metabolomic profiles can be used for classifying disease by type and stage, for prognosis, and for testing the effectiveness of therapeutics using statistical learning methods. For example, high-resolution magic angle spinning NMR (HRMAS–NMR), which can quantitatively identify a range of metabolites while leaving the tissue sample intact for further studies, has been used to profile and classify prostate tumors [39] and to detect drug efficacy in liposarcoma [40].

#### Biological images.

Advances in optics, digital detectors, and automation have significantly improved biological imaging technology and have led to a large increase of quantitative information extracted from digital images. Fluorescent and confocal microscopy, and whole-body imaging of model organisms [41,42] can be used to test specific hypotheses of cellular function and disease. Deep tissue–penetrating infrared light and various alterations of two-photon laser scanning microscopy (2PLSM) have been successfully used to reveal the dynamic nature and spatio–temporal aspects of hematopoietic tissue [43], organ development [44], and neurobiology [45]. Fluorescent proteins and photo bleaching techniques enable visualization of protein localization, protein–protein interactions, and protein fate in vivo [46]. In clinical settings, particularly for solid tumors, better resolution and higher contrast dyes have allowed the use of magnetic resonance imaging (MRI), computed tomography (CT) scan, positron emission tomography (PET) scan, and ultrasound for diagnosis and tracking disease progression. As imaging data is highly context-specific, data aggregation from multiple sources is possible only if sufficient metadata on samples, microscope, and data derivation algorithms are available.

#### Clinical data.

Clinical data is information about patients that is collected using surveys, during doctors' office visits, through administration of standard treatment procedures, or during clinical trials. Typical cancer clinical trials are conducted to determine the safety and efficacy of a drug in humans and depend on detailed patient information for accurate interpretation of results. Clinical trials range from pilot studies for feasibility assessment of the trial to more involved Phase I to IV trials [47]. Patient data collected during clinical trials includes family history (for example, if mother or sister had breast cancer), habits (for example, smoking/drinking), concomitant medications, alternate therapies, baseline characteristics preceding treatment, diagnostic parameters and clinical staging, treatment and procedural details, adverse events (toxicity), and clinical endpoints (for example disease recurrence or survival). For example, in solid tumors, tumor measurement is done at prespecified intervals for response assessment according to standards such as Response Evaluation Criteria in Solid Tumors (RECIST) [48,49]. Clinical data is then used in clinical research, for example, to relate exposure factors or treatment parameters to clinical outcome. As clinical data is often collected longitudinally at multiple visits, potentially by different health professionals, organization and storage of the data in standard formats is critical for analysis and interpretation.

###

**Box 1. Oncomine: A Case Study in Microarray Data Aggregation and Analysis**

The Oncomine cancer microarray database is an integrated meta-analysis platform that overcame diverse data integration and normalization challenges to enable comprehensive analysis of complex multistudy disease datasets [52,53,68]. A software pipeline was developed to parse gene expression data, raw or log-transformed, from native formats into numeric matrices of reporter rows and sample columns excluding study-specific normalization. Comprehensive mapping of probe identifiers (IDs) from oligonucleotide arrays or IMAGE clone IDs from cDNA arrays to a common Unigene build, Genbank accession numbers, and other commonly used database identifiers that link to gene annotation was also critical. Samples were renamed and reassigned using NCI nomenclature for consistency across studies. Lack of adherence of the individual datasets to any common standards, such as MIAME, complicated the data aggregation process. Another major hurdle was the complexity and non-uniformity of sample description information. For example, diverse representations of clinical sample description make it difficult to compare histological data, such as Gleason score for prostate tumors or Estrogen receptor status for breast carcinomas. This problem was addressed by mapping to a common data format using parameter/value pairs. Finally, each study was independently normalized and archived in a relational database. A large amount of software engineering work was required to deal with the structure and large size of gene expression data and provide a robust query and analysis tool.

Software platforms such as Oncomine are important for discovery and algorithm development. When mined using appropriate algorithms, such as the cancer outlier profile analysis (COPA) method, they can supplement experiments to make fundamental contributions to cancer genetics [69]. The challenges encountered during the creation of Oncomine emphasize the need for data representation standards and public data warehouses that transcend a single community's needs to allow for integrative studies. Statistical normalization and analysis methods for such integrated datasets are also required. Further enriching transcriptome data with complementary information, such as quantitative proteomic data, will present new challenges resulting from even higher data-dimensionality and volume, concordance and discordance between mRNA and corresponding protein data, and potential for information conflict, but also will provide new opportunities for discovery [70–72].

### Where Computational Biology Can Help

#### Data collection, organization, aggregation, and storage.

To effectively use genome-scale molecular information, it must be collected, organized in a standard way, aggregated, and stored so that it is widely accessible to the research community. Aggregation is pooling data from multiple experiments of the same type. The advantages of data aggregation are: it can increase the sample size and lead to improved statistical power for comparisons, and it can improve coverage, for instance, over more cell types, different parts of the tumor, or from different populations. Public biomedical data repositories that organize, aggregate, and store data from genome-scale molecular experiments are increasingly available for diverse data types (Tables 1–4).

Data from these repositories support comprehensive molecular analysis of tumors. For instance, commonly activated gene signatures [50], coordinately regulated gene modules involved in a biological process [51], and regulatory programs that control disease development [52,53] in various cancers have been identified by combining data from multiple microarray datasets. This dataset pooling or meta-analysis helps draw inferences that may not be possible with a single study with a limited number of samples/observations.

However, data aggregation is difficult unless standard methods for data collection, organization, archiving, and exchange are developed and followed. Table 5 summarizes some of the standards for different types of biological data. Development and community-wide use of standards enhances the ability of research groups to exchange data and provides a strong foundation on which to build data storage, processing, and analysis software.

**Table 5:**
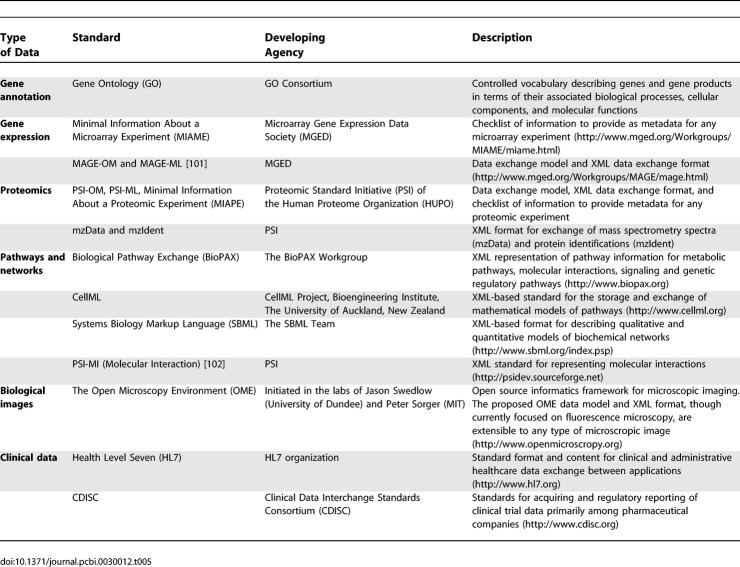
Data Standardization Efforts in Biomedical Research

In Box 1 we present a case study on Oncomine—a microarray data aggregation platform—highlighting the challenges encountered in aggregating microarray data from multiple sources and the opportunities that it provides.

#### Data integration.

Data integration is the combination of heterogeneous biological data encoded with different semantics. Integration of heterogeneous data is useful not only to validate and to improve confidence in experimental results but also to develop more complete models of biological systems. For instance, real time quantitative RT–PCR data are routinely used to validate cDNA array experiment results. Integration of gene expression and proteomics data, for example, could be used to identify post-transcriptional or post-translational modifications. It could also provide insights into the advantages and shortcomings of particular experimental methods.

The integration of diverse experimental data to build models of biological processes, or pathways, will boost our ability to identify clinical markers and therapeutic targets and to interpret genotype information. For instance, a marker such as prostate specific antigen (PSA) may be widely known and used clinically without much knowledge about its biology. Knowing the pathway involving the marker gene allows other pathway components, or the entire pathway, to serve as a more specialized marker.

Clinical data, securely and ethically accessed, can be integrated with molecular data from basic research to gain insight into disease state and lead to better treatments. Molecular and clinical data has been integrated for identification of clinically relevant subtypes of leukemia with 100% sensitivity and specificity [54]. Analysis of molecular profiles on biospecimens from patients before, during, and after therapy can lead to identification of drug-responsive or nonresponsive profiles that could be used to optimize choice of therapy. In the case of advanced non-small cell lung cancer (NSCLC), a significant difference in response to the kinase inhibitor gefitinib (Iressa) was observed for patients with mutations or amplifications in the *EGFR* gene [55,56]. Comparison of patient molecular profiles with poor and favorable outcomes can be used to predict disease outcome (prognosis). For example, in breast cancer patients, the estrogen-receptor status of the primary tumor and other clinical features have been used to construct nomograms that predict the likelihood of developing non-sentinel lymph node (non-SLN) metastases. Such information can be used to assess metastatic risk and the need for complete axillary lymph node dissection [57]. Additionally, a 32-gene expression signature distinguished p53-mutant and wild-type breast tumors of different histologies, and this strategy outperformed sequence-based assessments of p53 in predicting prognosis and therapeutic response [58]. Combination of molecular profiles with clinical profiles can also help select patients for targeted treatment.

Effective integration of heterogeneous data is difficult since important information necessary to decipher data semantics, such as context, could be missing. It is often possible for the human brain to infer this information using prior knowledge, but such tasks have remained impossible to encode into a model or rule-based computational procedure. Thus, missing information can lead to errors during integration. For example, a query may identify relevant datasets labeled with the term *metastasis*. However, metastatic processes could be different among tissues, so tissue information is required to avoid errors in dataset selection. Also, datasets labeled with alternate descriptions of metastasis may be missed in the search. As another example, proper mapping of genes and proteins between microarray and mass spectrometry data is an important requirement for integrating the datasets. This task could be confounded if the measured gene transcript is a different splice variant than the measured protein product. Thus, correct data integration requires semantic compatibility among datasets and context resolution.

In Box 2 we present two successful examples of how integration of heterogeneous data from different sources can help in biological discoveries. In the first example, publicly available data was used to help identify *LRPPRC* as a specific gene involved in Leigh syndrome, a complex hereditary disease. In the other example, the discovery of the central role of micropthalmia-associated transcription factor *(MITF)* amplification to malignant melanoma was accomplished using experimental and computational approaches to integrate copy number data, with publicly available gene expression and genome information. These examples provide proof of principle that there already exists a wealth of biological data in the public domain, and data integration approaches can be used to better understand biology and development of disease, including cancer.

####

**Box 2. Data Integration for Biological Discovery: Case Studies**

***LRPPRC* in Leigh syndrome.**

Data integration was applied to identify one of the genes responsible for Leigh syndrome [73]. Classical Leigh syndrome is an early onset fatal neurodegenerative disorder characterized by bilateral lesions in the brain stem, basal ganglia thalamus, and spinal cord and is known to involve a cytochrome c oxidase (COX) deficiency mapped to Chromosome 9 [74,75]. The French Canadian form of Leigh syndrome (LSFC) is distinct and is known to involve a gene on Chromosome 2, where no known cytochrome c oxidase gene is localized [76]. However, clinical and biochemical data suggest that a mitochondrial respiratory chain disorder is involved. From a genome-wide association study that mapped LSFC to Chromosome 2p16–21, Mootha et al. collected 15 known and 15 predicted genes using the UCSC genome browser. Microarray databases from the Whitehead, RIKEN array database and the Genomics Institute of the Novartis research foundation were then used to identify genes that coexpressed with known mitochondrial genes. *LRPPRC,* which mapped to the LSFC candidate genomic region, showed the highest correlated gene expression with known mitochondrial genes. *LRPPRC* was also found in a list of mitochondrial-associated proteins identified by mass spectrometry. Neither method alone was enough to implicate the specific gene. Thus, data integration overcame the incomplete coverage, low sensitivity, or specificity limitations of the individual experimental approaches.

***MITF* in malignant melanoma.**

Garraway et al. used an integrative approach to identify *MITF* as a “lineage survival” or “lineage addiction” oncogene required for development and maintenance of malignant melanoma [77]. The authors used the NCI60 panel, which is a collection of 59 human cancer cell lines derived from nine different types of tissues. SNP arrays of NCI60 cell lines were used to define genomic subclusters that were specifically amplified in the melanoma subset. This information was then integrated with the publicly available NCI60 gene expression data generated by the genomics institute of the Novartis foundation to correlate gene expression with the copy number gain. Remarkably, *MITF* was the only highly expressed gene in the amplicon identified in the SNP array analysis. This result was validated using FISH and automated quantitative analysis (AQUA) of MITF protein levels in patient samples. As before, public availability of gene expression data was instrumental for the authors to integrate expression data with their own SNP data in defining the function of a gene in the context of cancer.

#### Software systems and algorithms for data analysis.

The volume of the data generated by modern biomedical studies is too large to be processed by the human brain alone. Data storage, querying, and presentation software systems and computational algorithms are required for effective interpretation of large-scale experimental data. Automated methods are now available to find genes or pathways that are significantly differentially expressed using molecular profiles [59–63]. The GOALIE algorithm maps the temporal evolution of biological processes from time-course gene expression data [64]. An algorithm for general integration of heterogeneous data that differ in type and size has been proposed [65]. Further studies of pathways in the context of gene expression and complementary genome-scale data will lead to the discovery of new pathway components, some of which could be new vulnerable points and therapeutic targets [66].

Pathway simulations, requiring detailed cellular models, have been used in model organisms, such as Escherichia coli and budding yeast, to find pathway regulators and to design new experiments that test hypotheses about the function of the pathway [67]. Applying these methods to predict the result of a tumor-specific mutation in multicellular organisms using human pathway information is an important research direction and major challenge for computational biology.

Development of software systems that integrate diverse biomedical research data types promise to support the study of disease biology and development. For instance, the REMBRANDT (Repository for Molecular Brain Neoplasia Data) framework attempts to integrate clinical and molecular data from the Glioma Molecular Diagnostic Initiative (GMDI)—a collaborative effort of the NCI and the US National Institute of Neurological Disorders and Stroke (NINDS) (http://rembrandt.nci.nih.gov). REMBRANDT can be used to query and generate statistical reports across all component glioma (brain tumor) datasets.

Computational prediction of the biological effects of drugs based on structure–function relationships across many targets can help increase the success rate of clinical trials and may forewarn of possible adverse events associated with the small-molecule therapy. This strategy could also be used to develop drug combinations to target multiple vulnerable points to shut down tumor growth. ADME/Tox (absorption, distribution, metabolism, excretion, and toxicity) prediction based on molecular profiles can help eliminate candidate drugs that have unacceptable toxicity early in the drug discovery process and thus reduce the cost of drug development.

#### Social challenges for computational approaches.

Aggregating, integrating, and analyzing experimental data from multiple sources must overcome social as well as technical challenges. Critically, while archives of datasets from molecular studies are often publicly available, a public clinical counterpart remains largely unavailable due to patient privacy concerns. Securely providing de-identified patient data obtained with adequate patient consent, for example, as per the US Health Insurance Portability and Accountability Act (HIPAA) guidelines (http://www.hhs.gov/ocr/hipaa), is a viable solution.

Data collected from biological samples must be clearly annotated using standard representations, including descriptions of the sample and experimental conditions. Without such information data integration is significantly more difficult, inefficient, and error-prone. Effort must be spent to make data publicly available, to agree on and use community standards, and most importantly to make computational tools easy to use for biologists; these steps will significantly improve the effectiveness of translational cancer research. Computing infrastructure for facilitating data aggregation/integration can use either centralized systems wherein an investigator accesses a central computer system that holds all the data, or, alternatively, federated systems where an investigator sends a query and the system assembles pertinent information from where it exists. Two examples of research computer systems for data integration are caBIG and BIRN's cyber infrastructure. The Cancer Biomedical Informatics Grid (caBIG) is a network to enable sharing of data and software tools across individuals and cancer research institutions to improve the pace of innovations in cancer prevention and treatment (http://cabig.cancer.gov). The Biomedical Informatics Research Network (BIRN) is a distributed virtual community of shared resources that currently supports the sharing and analysis of neuroimaging data (http://www.nbirn.net).

### Concluding Remarks

Computational biology is pivotal for effectively using large and diverse data resources to provide insights into disease biology and to optimize treatment. Modeling and simulation techniques, standards, and software systems must be enhanced to deal with expanding molecular and clinical information. Making well-organized experimental datasets widely accessible will spur algorithm development, testing, and comparison, leading to the development of better computational methods. These new computational tools will allow us to effectively interpret available genome-scale datasets to improve disease diagnosis, prognosis, therapy, and prevention. 

## Abbreviations

caBIG: Cancer Biomedical Informatics Grid
CGH: comparative genomic hybridization
FISH: fluorescent in situ hybridization
mRNA: messenger RNA
miRNA: microRNA
MITF: micropthalmia-associated transcription factor
NCI: National Cancer Institute
SNP: single nucleotide polymorphism
